# Early risk stratification of adverse progression in pediatric *Mycoplasma pneumoniae* pneumonia using a multicenter CT radiomics model

**DOI:** 10.3389/fmed.2026.1905516

**Published:** 2026-08-20

**Authors:** Xiaoyu Yao, Zheng Liu

**Affiliations:** Department of Critical Care Medicine, Baoding First Central Hospital, Baoding, China

**Keywords:** computed tomography, machine learning, *Mycoplasma pneumoniae* pneumonia, plastic bronchitis, radiomics

## Abstract

**Objective:**

This study sought to establish and externally validate an interpretable CT-based radiomics framework for early prediction of adverse disease progression in children with *Mycoplasma pneumoniae* pneumonia (MPP).

**Methods:**

In this multicenter retrospective study, 419 children with MPP from three hospitals were assigned to training (*n* = 296), testing (*n* = 59), and validation (*n* = 64) cohorts. Adverse progression was defined as refractory MPP, plastic bronchitis, or both. Radiomics features were extracted from pulmonary lesions and selected using SelectKBest and least absolute shrinkage and selection operator (LASSO) regression. Three random forest models, namely the clinical-imaging, radiomics, and integrated models, were evaluated by receiver operating characteristic (ROC) analysis, calibration, decision curve analysis, and pairwise comparisons.

**Results:**

Among the 419 included children, 70 developed adverse progression. In the training cohort, D-dimer, white blood cell count, systemic immune-inflammation index, lobar atelectasis, and number of involved lung lobes were significantly associated with progression (all *p* < 0.05). In the external validation cohort, the integrated model (AUC, 0.828; 95% CI, 0.645–0.957) achieved an accuracy of 0.906, F1 score of 0.727, sensitivity of 0.727, and specificity of 0.943, outperforming the Clinical-Imaging model (AUC, 0.736; 95% CI, 0.514–0.913; *p* = 0.013) and radiomics model (AUC, 0.813; 95% CI, 0.628–0.950; *p* = 0.043).

**Conclusion:**

An integrated model incorporating inflammatory indices, CT findings, and radiomics features showed promise for early risk stratification for adverse progression in pediatric MPP.

## Introduction

The recent re-emergence of *Mycoplasma pneumoniae* infections after the COVID-19 pandemic has refocused attention on pediatric *Mycoplasma pneumoniae* pneumonia (MPP) as a major public health and clinical challenge (1). Although MPP has traditionally been regarded as a mild or self-limited cause of community-acquired pneumonia, hospitalized children may experience substantial disease burden, prolonged symptoms, radiological progression, and severe extrapulmonary or airway complications (1, 2). This diagnostic uncertainty is particularly important because early clinical manifestations are often nonspecific, whereas delayed recognition of progressive disease may reduce the window for timely monitoring, bronchoscopy, anti-inflammatory therapy, or individualized antimicrobial adjustment (3, 4).

The clinical progression of pediatric MPP reflects both pathogen-related injury and dysregulated host responses. Refractory *Mycoplasma pneumoniae* pneumonia (RMPP) and plastic bronchitis (PB) are severe but overlapping complications, involving persistent inflammation (2–4), coagulation activation, mucus plugging, fibrin deposition, airway obstruction, and parenchymal injury (5–8). Routine inflammatory and coagulation markers, including lactate dehydrogenase, C-reactive protein, ferritin, neutrophil-related indices, and D-dimer, have been associated with severe or refractory MPP and are readily available at admission (3, 9, 10). However, laboratory indicators alone cannot capture pulmonary lesion burden or spatial heterogeneity. Chest computed tomography (CT) provides complementary anatomical information, but visual CT features such as consolidation, atelectasis, multilobar involvement, and pleural effusion remain partly subjective and may miss subtle quantitative differences within lesions (11, 12).

Radiomics converts routine medical images into high-dimensional quantitative features describing lesion intensity, shape, texture, and spatial organization, thereby complementing visual assessment in precision risk prediction (13, 14). Recent studies have applied CT-based radiomics or machine learning to predict RMPP, delayed radiological recovery, and PB in children with MPP (15–17). However, existing models often focus on a single adverse outcome, use variables obtained later in the disease course, lack independent external validation, or provide limited interpretability, restricting their value for early clinical decision support. Thus, radiomics may be most useful when integrated with admission clinical and laboratory information in an externally validated and interpretable framework.

To address these gaps, the present multicenter retrospective study aimed to develop and externally validate an early, interpretable risk stratification framework for adverse progression in children with MPP. Adverse progression was defined as the occurrence of RMPP, PB, or both during the disease course. We hypothesized that integrating inflammatory markers, conventional CT manifestations, and radiomics features would provide a more robust and clinically meaningful framework for early identification of children at risk for adverse MPP progression.

## Materials and methods

### Study design and participants

This retrospective study was conducted in accordance with the Declaration of Helsinki and received approval from the Ethics Committee of all participating centers (Approval No. HDFYLL-KY-2024-002). The requirement for informed consent from patients or their legal guardians was waived owing to the retrospective nature of the study.

**Figure 1:**
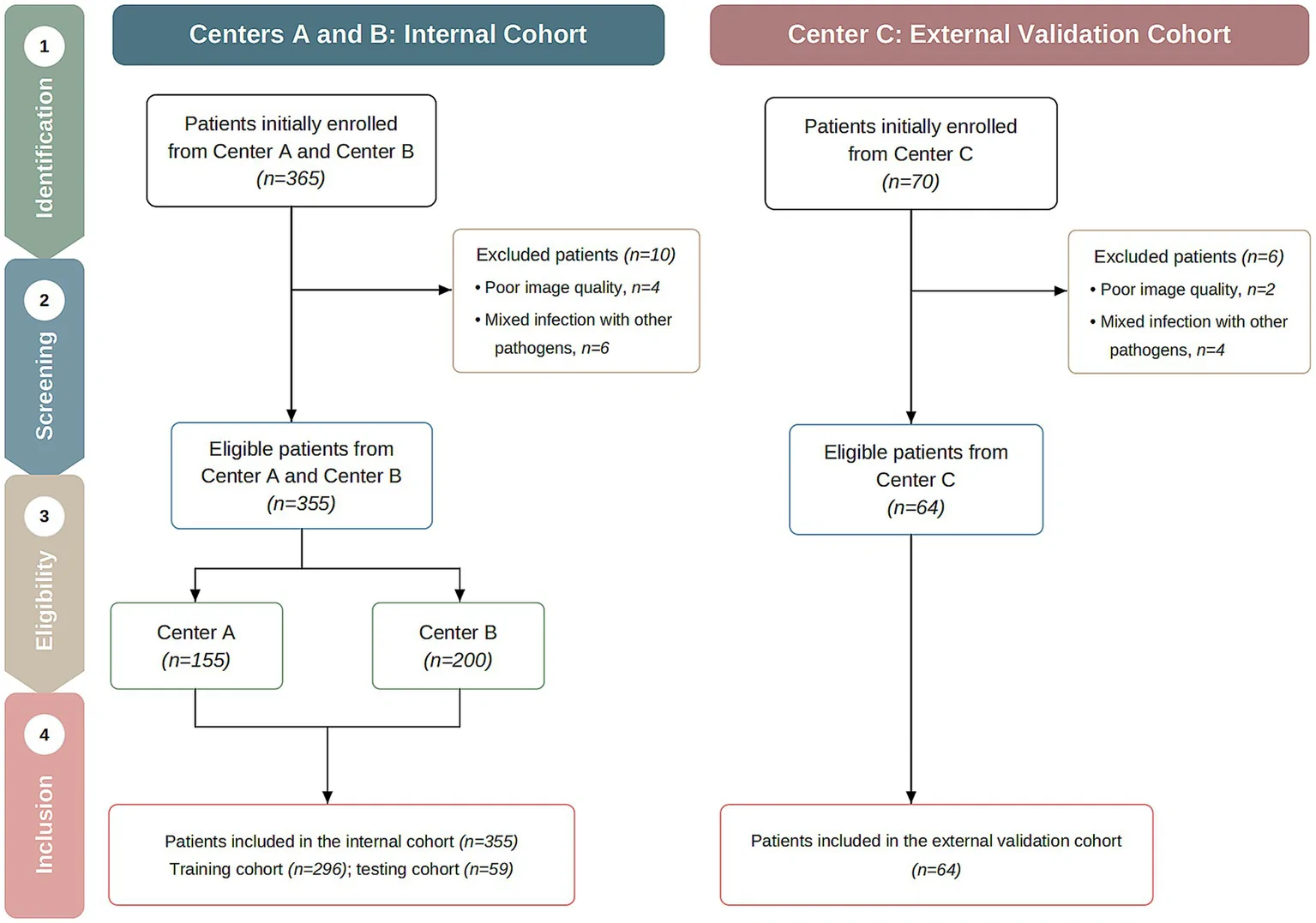
Patient selection and cohort allocation for the multicenter study.

Demographic information, laboratory results, and imaging examinations were retrospectively retrieved from three participating hospitals between July 2024 and July 2025. Data from two institutions were pooled and randomly divided into training and testing cohorts at an 8:2 ratio using stratified random sampling according to adverse progression status. Cases collected from the third institution were reserved as an independent external validation set for assessment of model robustness and generalizability (Figure 1).

Children aged 1 month to 18 years were eligible for inclusion if they met the diagnostic criteria for MPP, which required: (1) compatible clinical manifestations of pneumonia; (2) radiological evidence of pulmonary infection; and (3) laboratory confirmation of *Mycoplasma pneumoniae* infection, defined by at least one of the following criteria: a positive *Mycoplasma pneumoniae* nucleic acid test, a single serum *Mycoplasma pneumoniae* antibody titer ≥1:160, or a fourfold or greater increase in paired serum antibody titers. Patients were excluded if they met any of the following criteria: (1) the presence of underlying diseases that could potentially affect disease progression or immune status, including immunodeficiency, chronic respiratory disease, congenital heart disease, renal disease, autoimmune disorders, malnutrition, endocrine disorders, or inherited metabolic diseases; (2) co-infection with other respiratory pathogens; (3) incomplete clinical, laboratory, or imaging data; or (4) a history of thoracic surgery.

The baseline time point was hospital admission, and only variables collected within 24 h of admission were analyzed to enable early risk assessment and avoid information leakage. The primary endpoint was any adverse progression, defined as RMPP, PB, or both. This composite endpoint was used as a pragmatic indicator of clinical deterioration rather than implying pathophysiological equivalence. RMPP reflects persistent nonresponse despite treatment, whereas PB is an airway obstruction confirmed by bronchoscopy. Although distinct, they may occur separately or together and both require closer monitoring or treatment escalation. The endpoint aimed to identify children at risk of clinically significant complications at admission. Outcomes were further categorized as RMPP only, PB only, or concurrent RMPP/PB for exploratory analysis.

RMPP was diagnosed according to established clinical criteria as persistent fever accompanied by worsening clinical manifestations and/or radiological findings despite appropriate macrolide therapy for at least 7 days. PB was diagnosed by bronchoscopic visualization and removal of bronchial casts causing airway obstruction, with histopathological confirmation of mucin–fibrin components when available (18).

### Clinical data collection and CT imaging assessment

Clinical and laboratory data were retrospectively collected from the electronic medical records and imaging archive systems of the participating centers. Recorded variables included sex, age, and fever grade. Laboratory parameters obtained within 24 h after admission included white blood cell count (WBC), absolute neutrophil count (NEUT), platelet count (PLT), creatine kinase-MB (CK-MB), lactate dehydrogenase (LDH), activated partial thromboplastin time (APTT), fibrinogen (FIB), D-dimer, C-reactive protein (CRP), neutrophil-to-lymphocyte ratio (NLR), platelet-to-lymphocyte ratio (PLR), and systemic immune-inflammation index (SII), which was calculated as PLT × NEUT / lymphocyte count. SII was entered into the models as an untransformed continuous variable, without a predefined cutoff, categorization, logarithmic transformation, or standardization.

Chest CT was not routinely performed or mandated for this study. At the participating centers, CT was obtained only when clinically indicated according to the treating team’s assessment and institutional pediatric imaging protocols. For this analysis, the baseline CT was defined as the earliest clinically acquired chest CT available during the disease episode. When multiple CT examinations were available, the earliest scan was selected as the baseline CT. Images were independently reviewed by two experienced pediatric radiologists (6 and 8 years of experience) who were blinded to clinical information and outcomes, with disagreements resolved by a senior radiologist (12 years of experience). Conventional CT features assessed included lobar atelectasis, consolidation pattern, consolidation mixed with ground-glass opacity, adjacent pleural thickening, pleural effusion, mediastinal lymph node enlargement, air bronchogram sign, interlobular septal thickening, reticular pattern, fiber cord, and number of involved lung lobes.

### CT image acquisition, preprocessing, and lesion segmentation

All patients were scanned in the supine position, and the scan range extended from the thoracic inlet to the costophrenic angles. CT images were acquired at end-inspiration after standardized breathing instructions to improve image quality and reduce motion artifacts. Chest CT examinations were performed using scanner systems available at the participating institutions. Detailed scanner information and acquisition parameters are provided in the Supplementary Table S1.

All images underwent standardized preprocessing, including resampling to isotropic voxels (1 × 1 × 1 mm^3^) using B-spline interpolation and intensity normalization to reduce inter-scanner variability and improve feature comparability across cohorts. Regions of interest were defined as all visible pulmonary lesion areas on baseline chest CT images. Manual segmentation was performed using ITK-SNAP software (version 4.0.0; http://www.itksnap.org/pmwiki/pmwiki.php). Lesion delineation was independently conducted by two radiologists, and discrepancies were reviewed and resolved by consensus with a senior radiologist with more than 15 years of experience in thoracic imaging. Inter-observer segmentation reproducibility was assessed in a random subset of 84 patients (approximately 20% of the full cohort), including cases from all three participating centers. Two radiologists independently delineated the pulmonary lesions in this subset, and patient-level agreement was quantified using the Dice similarity coefficient, yielding a mean value of 0.87 ± 0.04. Three-dimensional regions of interest were manually delineated slice by slice on baseline chest CT images. All visible inflammatory pulmonary lesions, including consolidation, ground-glass opacity, and atelectatic areas related to MPP, were included. Large vessels, normal bronchi, pleural effusion, and extrapulmonary structures were carefully excluded. The final consensus segmentation was used for radiomics feature extraction.

### Radiomics feature extraction and selection

Radiomics features were extracted from the segmented pulmonary lesions on baseline CT images using the PyRadiomics package (version 3.0) (19). Feature extraction was performed on both original and filtered images. Fifteen image filters were applied, as detailed in the Supplementary Table S2, yielding a total of 1,904 radiomics features for each patient.

The extracted features covered seven categories: first-order statistical features (*n* = 378), shape features (*n* = 14), gray-level co-occurrence matrix features (GLCM; *n* = 441), gray-level run-length matrix features (GLRLM; *n* = 336), gray-level size-zone matrix features (GLSZM; *n* = 336), gray-level dependence matrix features (GLDM; *n* = 294), and neighboring gray-tone difference matrix features (NGTDM; *n* = 105).

Radiomics feature selection was conducted only in the training cohort to avoid information leakage from the testing and external validation cohorts. First, the SelectKBest method was used to rank features according to their univariable relevance to any adverse progression, and the top 50% of features were retained. Subsequently, least absolute shrinkage and selection operator (LASSO) regression was applied to further reduce dimensionality, remove redundant features, and identify the final subset of radiomics predictors for model construction.

### Model construction

**Figure 2:**
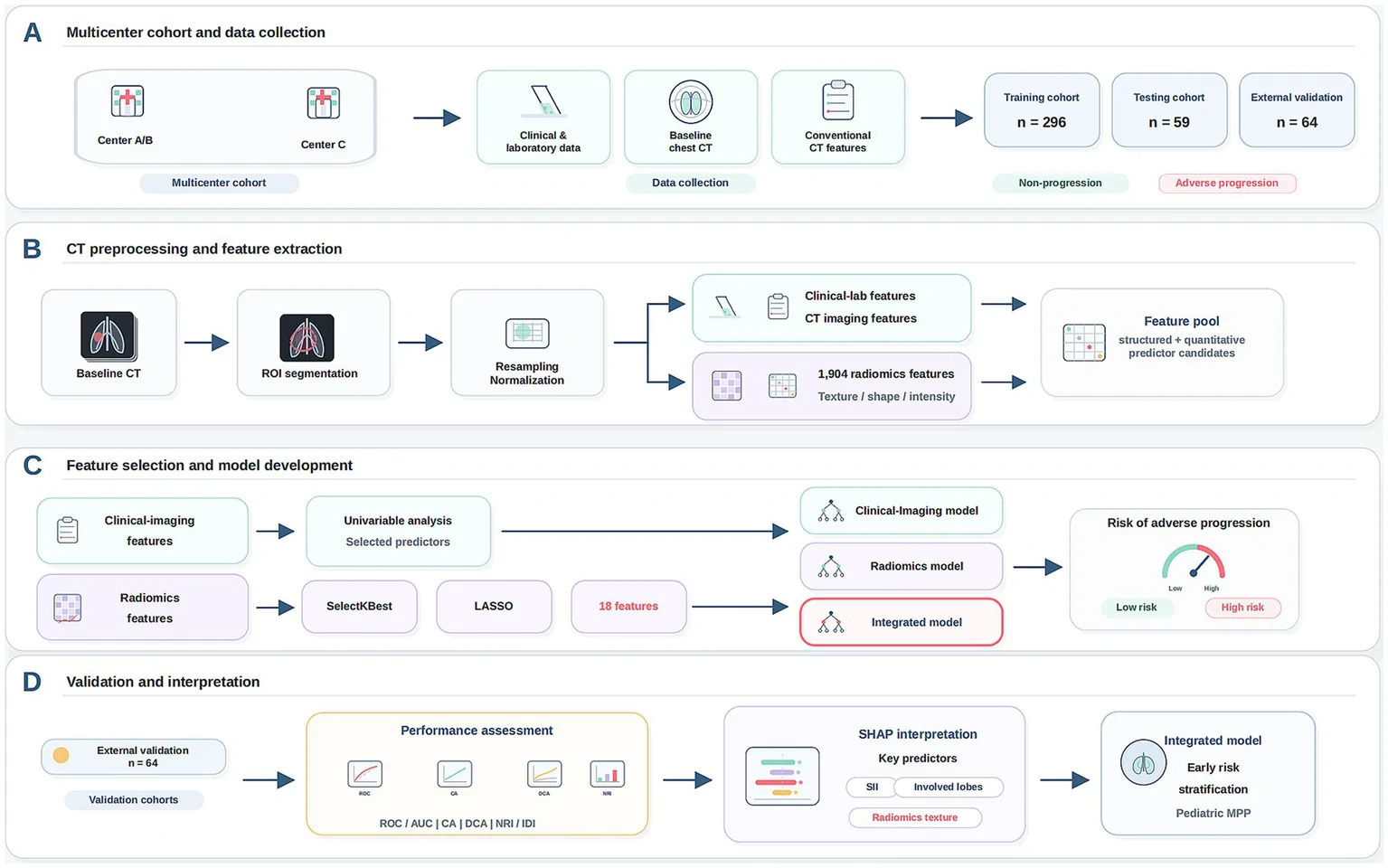
Overall workflow of model development and validation. **(A)** Multicenter cohort construction and data collection. **(B)** CT preprocessing, ROI segmentation, and feature extraction. **(C)** Feature selection and development of the Clinical-Imaging, Radiomics, and integrated models. **(D)** External validation, performance assessment, SHAP-based interpretation, and early risk stratification of adverse progression in pediatric MPP. ROI, region of interest; CT, computed tomography; LASSO, least absolute shrinkage and selection operator; ROC, receiver operating characteristic; AUC, area under the curve; CA, calibration analysis; DCA, decision curve analysis; NRI, net reclassification improvement; IDI, integrated discrimination improvement; SHAP, Shapley additive explanations; SII, systemic immune-inflammation index; MPP, *Mycoplasma pneumoniae* pneumonia.

Univariable logistic regression was performed in the training cohort to identify clinical, laboratory, and conventional CT variables associated with any adverse progression, and variables with *p* < 0.05 were retained as candidate predictors. Subsequently, three random forest models were developed: a Clinical-Imaging model based on selected clinical, laboratory, and CT features, a radiomics model based on the selected radiomics features, and an integrated model combining both clinical-imaging and radiomics information. Model training was conducted in the training cohort, with performance subsequently assessed in the testing and external validation cohorts. The overall study workflow is illustrated in Figure 2.

Random forest classifiers were implemented in Python using scikit-learn version 1.5.1. All feature selection and hyperparameter tuning were performed exclusively in the training cohort to avoid information leakage. Hyperparameters were optimized using grid search with stratified five-fold cross-validation, with AUC as the primary optimization metric; the detailed search space is provided in Supplementary Table S3. Class imbalance was addressed using class-weighted random forest models. A fixed probability threshold of 0.50 was used to convert predicted probabilities into binary classifications for the calculation of accuracy, sensitivity, specificity, precision, and F1 score. This threshold was applied unchanged to the training, testing, and external validation cohorts. The baseline predictor dataset was finalized before model development. All feature selection and hyperparameter optimization were performed using baseline predictors and outcome labels from the training cohort only. Neither the predictors nor the outcome labels from the testing and external validation cohorts were used during model development.

### Statistical analysis

Continuous variables are presented as medians with interquartile ranges, and categorical variables as frequencies and percentages. Group comparisons were performed using Pearson’s chi-square test or Fisher’s exact test for categorical variables and the Mann–Whitney U test or Kruskal–Wallis test for continuous variables, as appropriate.

Univariable logistic regression was used to identify clinical, laboratory, and CT variables predictors of adverse progression. Model performance was assessed using receiver operating characteristic (ROC) curves, AUC, accuracy, sensitivity, specificity, precision, and F1 score. Calibration curves and decision curve analysis (DCA) evaluated calibration and clinical utility. Model comparisons were conducted using DeLong’s test, McNemar’s test, net reclassification improvement (NRI), and integrated discrimination improvement (IDI). An exploratory component-level analysis was conducted using a fixed probability threshold of 0.50 to assess model performance across outcome phenotypes. Sensitivity and exact 95% confidence intervals were calculated using the Clopper–Pearson method, and results were reported descriptively without formal statistical comparisons due to small sample sizes. Shapley Additive Explanations (SHAP) were used to interpret the integrated model. Analyses were performed in R 4.3.2 and Python (scikit-learn 1.5.1). A two-sided *p* < 0.05 was considered statistically significant.

## Results

### Patient characteristics

A total of 419 children with MPP were enrolled, including 296, 59, and 64 patients in the training, testing, and external validation cohorts, respectively (Figure 1). The training cohort (149 males; age, 7.162 ± 3.214 years), testing cohort (25 males; age, 7.068 ± 3.681 years), and external validation cohort (35 males; age, 7.203 ± 3.725 years) showed no significant differences in sex distribution or age (both *p* > 0.05).

Among the 70 patients who experienced adverse progression, 19 presented with RMPP only, 8 with PB only, and 43 with concurrent RMPP and PB. In the training cohort, 13 patients had RMPP only, 6 had PB only, and 30 had concurrent RMPP and PB. The respective counts were 3, 1, and 6 in the testing cohort and 3, 1, and 7 in the external validation cohort, respectively.

Across all three cohorts, significant differences between the non-progression and progression groups were consistently observed for SII, lobar atelectasis, and the number of involved lung lobes, with significant differences identified in the training (*p* = 0.018, *p* < 0.001, and *p* < 0.001, respectively), testing (*p* = 0.031, *p* = 0.005, and *p* = 0.002), and external validation cohorts (*p* = 0.024, *p* = 0.005, and *p* = 0.003; Table 1). Across the three cohorts, CK-MB and fibrinogen differed significantly among cohorts (*p* = 0.025 and *p* = 0.045, respectively), whereas other baseline clinical, laboratory, and conventional CT characteristics showed no significant inter-cohort differences (Table 1).

**Table 1:** Baseline clinical, laboratory, and CT characteristics of children with MPP stratified by subsequent adverse progression status.

Characteristics	Training cohort (*n* = 296)			Testing cohort (*n* = 59)			Validation cohort (*n* = 64)			Overall (*n* = 419)
	Non-progression	Progression	*P*-Intra value	Non-progression	Progression	*P*-Intra value	Non-progression	Progression	*P*-Intra value	*P*-Inter value
	(*n* = 247)	(*n* = 49)		(*n* = 49)	(*n* = 10)		(*n* = 53)	(*n* = 11)		
Male	123 (49.8%)	26 (53.1%)	0.676	23 (46.9%)	2 (20.0%)	0.166	29 (54.7%)	6 (54.5%)	1.000	0.378
Age, years	7.000 (5.000, 9.000)	7.000 (5.000, 9.000)	0.901	7.000 (5.000, 9.000)	5.500 (4.250, 7.000)	0.470	7.000 (4.000, 9.000)	7.000 (6.000, 8.000)	0.727	0.789
Type of fever			0.028			0.485			0.970	0.859
Absent	27 (10.9%)	5 (10.2%)		5 (10.2%)	1 (10.0%)		6 (11.3%)	1 (9.1%)		
Low-grade	77 (31.2%)	12 (24.5%)		17 (34.7%)	2 (20.0%)		11 (20.8%)	3 (27.3%)		
Mid-grade	123 (49.8%)	21 (42.9%)		24 (49.0%)	5 (50.0%)		31 (58.5%)	6 (54.5%)		
Hyperpyrexia	20 (8.1%)	11 (22.4%)		3 (6.1%)	2 (20.0%)		5 (9.4%)	1 (9.1%)		
WBC, ×10⁹/L	7.660 (6.040, 9.945)	8.980 (6.480, 12.160)	0.017	7.860 (6.290, 9.850)	7.910 (6.268, 11.595)	0.785	8.800 (6.830, 10.310)	8.640 (6.770, 11.250)	0.901	0.455
NEUT, ×10⁹/L	4.690 (3.255, 6.465)	4.990 (3.380, 6.840)	0.194	4.570 (3.340, 6.170)	5.020 (3.595, 7.330)	0.505	4.860 (3.930, 7.110)	5.200 (3.420, 7.480)	0.929	0.299
PLT, ×10⁹/L	280.000 (230.000, 370.500)	313.000 (248.000, 372.000)	0.238	316.000 ± 95.256	280.600 ± 99.615	0.322	280.000 (223.000, 347.000)	308.000 (253.500, 323.500)	0.742	0.784
CK-MB, ng/mL	2.240 (1.560, 2.690)	1.700 (1.100, 2.860)	0.204	2.206 ± 0.945	1.550 ± 0.925	0.062	1.710 (0.900, 2.380)	1.810 (1.110, 2.140)	0.755	0.025
LDH, U/L	272.000 (232.000, 323.000)	277.000 (215.000, 329.000)	0.486	281.000 (234.000, 338.000)	260.000 (221.500, 374.500)	0.754	264.000 (229.000, 335.000)	301.000 (205.500, 347.000)	0.993	0.714
APTT, s	33.000 (30.700, 35.400)	33.100 (30.600, 35.700)	0.991	33.200 (31.400, 35.300)	31.400 (30.150, 33.650)	0.079	32.983 ± 3.370	34.027 ± 4.857	0.509	0.846
FIB, g/L	3.810 (3.310, 4.290)	3.890 (3.410, 4.360)	0.327	3.910 (3.580, 4.290)	4.140 (3.797, 4.630)	0.342	4.010 (3.650, 4.610)	3.810 (3.220, 4.480)	0.482	0.045
D-dimer, ng/mL	0.305 (0.175, 1.097)	137.000 (0.190, 289.000)	0.001	0.270 (0.152, 0.904)	6.115 (0.252, 137.000)	0.100	0.485 (0.212, 137.000)	0.518 (0.224, 183.500)	0.749	0.405
CRP, mg/L	6.150 (1.870, 15.575)	6.940 (2.270, 19.120)	0.215	6.110 (2.340, 12.030)	4.325 (0.847, 28.583)	0.694	8.220 (3.860, 18.640)	20.290 (4.820, 33.840)	0.355	0.191
NLR	2.273 (1.333, 3.440)	2.012 (1.493, 3.572)	0.779	2.036 (1.374, 2.911)	1.995 (1.806, 3.088)	0.348	2.522 (1.723, 3.548)	2.808 (1.524, 4.009)	0.873	0.149
PLR	138.057 (104.119, 174.184)	132.932 (87.205, 159.278)	0.242	129.752 (98.940, 186.066)	128.024 (98.134, 167.316)	0.912	139.908 (104.455, 192.697)	151.630 (112.518, 212.168)	0.803	0.780
SII, ×10⁹/L	560.912 (434.139, 866.525)	635.364 (396.774, 1006.572)	0.018	551.445 (427.555, 885.914)	573.082 (387.259, 864.684)	0.031	655.419 (439.371, 1150.541)	819.110 (455.626, 1238.338)	0.024	0.336
Lobar atelectasis			<0.001			0.005			0.005	0.961
Absent	222 (89.9%)	33 (67.3%)		45 (91.8%)	5 (50.0%)		49 (92.5%)	6 (54.5%)		
Present	25 (10.1%)	16 (32.7%)		4 (8.2%)	5 (50.0%)		4 (7.5%)	5 (45.5%)		
Consolidation pattern			0.106			0.379			0.328	0.515
Absent	49 (19.8%)	5 (10.2%)		10 (20.4%)	3 (30.0%)		12 (22.6%)	2 (18.2%)		
Patchy	64 (25.9%)	9 (18.4%)		16 (32.7%)	2 (20.0%)		14 (26.4%)	1 (9.1%)		
Segmental	65 (26.3%)	20 (40.8%)		13 (26.5%)	1 (10.0%)		8 (15.1%)	4 (36.4%)		
Wedge-shaped	69 (27.9%)	15 (30.6%)		10 (20.4%)	4 (40.0%)		19 (35.8%)	4 (36.4%)		
Consolidation mixed GGO			0.317			0.731			0.526	0.729
Absent	115 (46.6%)	19 (38.8%)		24 (49.0%)	6 (60.0%)		23 (43.4%)	6 (54.5%)		
Present	132 (53.4%)	30 (61.2%)		25 (51.0%)	4 (40.0%)		30 (56.6%)	5 (45.5%)		
Adjacent pleura thickening			0.260			1.000			0.438	0.798
Absent	237 (96.0%)	45 (91.8%)		45 (91.8%)	10 (100.0%)		51 (96.2%)	10 (90.9%)		
Present	10 (4.0%)	4 (8.2%)		4 (8.2%)	0 (0.0%)		2 (3.8%)	1 (9.1%)		
Pleural effusion			0.513			0.538			0.438	0.458
Absent	235 (95.1%)	45 (91.8%)		45 (91.8%)	9 (90.0%)		51 (96.2%)	10 (90.9%)		
Mild	9 (3.6%)	2 (4.1%)		1 (2.0%)	1 (10.0%)		2 (3.8%)	1 (9.1%)		
Moderate	2 (0.8%)	1 (2.0%)		1 (2.0%)	0 (0.0%)		0 (0.0%)	0 (0.0%)		
Severe	1 (0.4%)	1 (2.0%)		2 (4.1%)	0 (0.0%)		0 (0.0%)	0 (0.0%)		
Mediastinal enlargement of lymph nodes			0.698			1.000			0.539	0.728
Absent	236 (95.5%)	48 (98.0%)		46 (93.9%)	10 (100.0%)		50 (94.3%)	10 (90.9%)		
Present	11 (4.5%)	1 (2.0%)		3 (6.1%)	0 (0.0%)		3 (5.7%)	1 (9.1%)		
Air bronchogram sign			0.413			1.000			0.314	0.231
Absent	96 (38.9%)	16 (32.7%)		23 (46.9%)	5 (50.0%)		19 (35.8%)	2 (18.2%)		
Present	151 (61.1%)	33 (67.3%)		26 (53.1%)	5 (50.0%)		34 (64.2%)	9 (81.8%)		
Interlobular septal thickening			0.928			0.676			1.000	0.771
Absent	183 (74.1%)	36 (73.5%)		39 (79.6%)	7 (70.0%)		40 (75.5%)	9 (81.8%)		
Present	64 (25.9%)	13 (26.5%)		10 (20.4%)	3 (30.0%)		13 (24.5%)	2 (18.2%)		
Reticular pattern			0.797			0.338			0.188	0.445
Absent	221 (89.5%)	45 (91.8%)		44 (89.8%)	8 (80.0%)		43 (81.1%)	11 (100.0%)		
Present	26 (10.5%)	4 (8.2%)		5 (10.2%)	2 (20.0%)		10 (18.9%)	0 (0.0%)		
Fiber cords			0.517			0.204			0.436	0.896
Absent	192 (77.7%)	36 (73.5%)		40 (81.6%)	6 (60.0%)		41 (77.4%)	10 (90.9%)		
Present	55 (22.3%)	13 (26.5%)		9 (18.4%)	4 (40.0%)		12 (22.6%)	1 (9.1%)		
Number of lobes involved			<0.001			0.002			0.003	0.999
1	120 (48.6%)	7 (14.3%)		24 (49.0%)	1 (10.0%)		25 (47.2%)	1 (9.1%)		
2	70 (28.3%)	8 (16.3%)		15 (30.6%)	1 (10.0%)		16 (30.2%)	1 (9.1%)		
3	30 (12.1%)	12 (24.5%)		6 (12.2%)	3 (30.0%)		6 (11.3%)	3 (27.3%)		
4	15 (6.1%)	10 (20.4%)		3 (6.1%)	2 (20.0%)		3 (5.7%)	2 (18.2%)		
5	12 (4.9%)	12 (24.5%)		1 (2.0%)	3 (30.0%)		3 (5.7%)	4 (36.4%)		

MPP, *Mycoplasma pneumoniae* pneumonia; CT, computed tomography; WBC, white blood cell count; NEUT, neutrophil count; PLT, platelet count; CK-MB, creatine kinase-MB; LDH, lactate dehydrogenase; APTT, activated partial thromboplastin time; FIB, fibrinogen; CRP, C-reactive protein; NLR, neutrophil-to-lymphocyte ratio; PLR, platelet-to-lymphocyte ratio; SII, systemic immune-inflammation index; GGO, ground-glass opacity.

### Selection of candidate predictors for model development

In the training cohort, among laboratory variables, higher D-dimer levels (OR = 2.209, 95% CI: 1.581–3.087, *p* < 0.001), WBC counts (OR = 1.391, 95% CI: 1.058–1.827, *p* = 0.018), and SII values (OR = 1.529, 95% CI: 1.174–1.992, *p* = 0.002) were all significantly associated with an increased risk of any adverse progression (Table 2). For conventional CT imaging characteristics, lobar atelectasis (OR = 4.717, 95% CI: 2.298–9.682, *p* < 0.001) and number of involved lung lobes (*p* < 0.001) were significantly associated with any adverse progression (Table 2). After radiomics feature selection, 18 radiomics features were retained and incorporated into subsequent radiomics model construction.

**Table 2:** Associations of early clinical, laboratory, and conventional CT features with any adverse progression in children with MPP.

Predictor	Coefficient	OR (95% CI)	*p*-value
Male	0.131	1.140 (0.617–2.106)	0.677
Age	−0.007	0.993 (0.730–1.349)	0.963
D-dimer	0.793	2.209 (1.581–3.087)	<0.001
WBC	0.330	1.391 (1.058–1.827)	0.018
Type of fever			0.056
Low-grade	−0.172	0.842 (0.271–2.609)	0.765
Mid-grade	−0.081	0.922 (0.319–2.663)	0.881
Hyperpyrexia	1.089	2.970 (0.890–9.910)	0.077
FIB	0.236	1.266 (0.964–1.661)	0.090
Consolidation pattern			0.101
Patchy	0.321	1.378 (0.434–4.373)	0.586
Segmental	1.104	3.015 (1.058–8.598)	0.039
Wedge-shaped	0.756	2.130 (0.726–6.251)	0.168
CRP	0.211	1.235 (0.960–1.589)	0.101
NEUT	0.191	1.211 (0.917–1.598)	0.177
Adjacent pleura thickening	0.745	2.107 (0.633–7.012)	0.225
Number of lobes involved			<0.001
1	−2.429	0.088 (0.001–5.410)	0.248
2	2.148	8.570 (0.403–182.186)	0.168
3	1.004	2.728 (0.118–62.969)	0.531
4	1.676	5.343 (0.222–128.466)	0.302
5	1.696	5.453 (0.240–124.124)	0.287
LDH	−0.201	0.818 (0.578–1.159)	0.259
CK-MB	−1.950	0.142 (0.003–6.322)	0.314
Consolidation mixed GGO	0.319	1.376 (0.735–2.574)	0.319
PLR	−0.263	0.768 (0.448–1.317)	0.338
Mediastinal enlargement of lymph nodes	−0.805	0.447 (0.056–3.544)	0.446
Air bronchogram sign	0.271	1.311 (0.685–2.511)	0.414
fiber cords	0.232	1.261 (0.625–2.542)	0.518
Reticular pattern	−0.280	0.756 (0.251–2.271)	0.618
SII	0.425	1.529 (1.174–1.992)	0.002
Pleural effusion			0.624
Mild	0.149	1.160 (0.243–5.551)	0.852
Moderate	0.960	2.611 (0.232–29.411)	0.437
Severe	1.653	5.222 (0.321–85.036)	0.246
Lobar atelectasis	1.551	4.717 (2.298–9.682)	<0.001
PLT	0.034	1.035 (0.788–1.360)	0.805
NLR	0.027	1.027 (0.760–1.388)	0.861
APTT	−0.018	0.982 (0.722–1.335)	0.907
Interlobular septal thickening	0.032	1.033 (0.515–2.069)	0.928

OR, odds ratio; CI, confidence interval; WBC, white blood cell count; NEUT, neutrophil count; PLT, platelet count; CK-MB, creatine kinase-MB; LDH, lactate dehydrogenase; APTT, activated partial thromboplastin time; FIB, fibrinogen; CRP, C-reactive protein; NLR, neutrophil-to-lymphocyte ratio; PLR, platelet-to-lymphocyte ratio; SII, systemic immune-inflammation index; GGO, ground-glass opacity.

### Predictive performance of the risk stratification models

**Figure 3:**
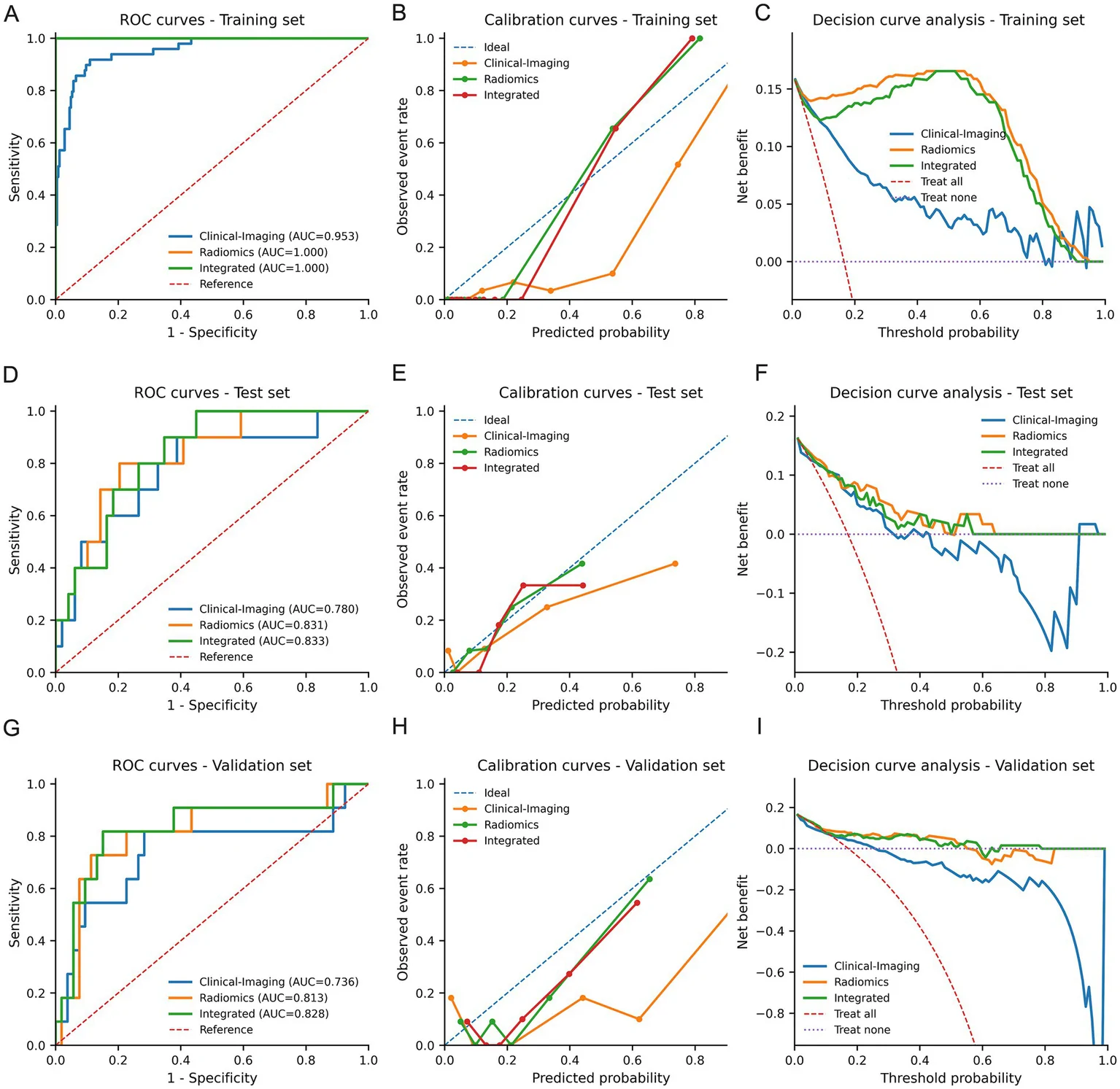
Performance of the three models for predicting adverse progression in children with MPP. **(A–C)** ROC, calibration, and decision curve analyses in the training cohort; **(D–F)** corresponding analyses in the test cohort; and **(G–I)** corresponding analyses in the external validation cohort. AUC, area under the curve.

The ROC curves, calibration curves, and decision curves of the radiomics, clinical-imaging, and integrated models for predicting any adverse progression are shown in Figure 3, and the corresponding performance metrics are summarized in Table 3. In the external validation cohort, the integrated model obtained an AUC of 0.828 (95% CI: 0.645–0.957), accuracy of 0.906, F1 score of 0.727, precision of 0.727, sensitivity of 0.727, and specificity of 0.943. The radiomics model achieved an AUC of 0.813 (95% CI: 0.628–0.950), accuracy of 0.875, F1 score of 0.636, precision of 0.636, sensitivity of 0.636, and specificity of 0.925. The clinical-imaging model showed an AUC of 0.736 (95% CI: 0.514–0.913), accuracy of 0.719, F1 score of 0.471, precision of 0.348, sensitivity of 0.727, and specificity of 0.717.

**Table 3:** Predictive performance of radiomics, clinical-imaging, and integrated models for early identification of any adverse progression in children with MPP.

Model	AUC (95% CI)	Accuracy	F1 score	Precision	Sensitivity	Specificity
Training cohort						
Radiomics	1.000 (1.000–1.000)	1.000	1.000	1.000	1.000	1.000
Clinical-imaging	0.953 (0.924–0.978)	0.865	0.692	0.556	0.918	0.854
Integrated	1.000 (1.000–1.000)	1.000	1.000	1.000	1.000	1.000
Testing cohort						
Radiomics	0.831 (0.668–0.949)	0.831	0.286	0.500	0.200	0.959
Clinical-imaging	0.780 (0.591–0.927)	0.797	0.455	0.417	0.500	0.857
Integrated	0.833 (0.701–0.942)	0.847	0.308	0.667	0.200	0.980
Validation cohort						
Radiomics	0.813 (0.628–0.950)	0.875	0.636	0.636	0.636	0.925
Clinical-imaging	0.736 (0.514–0.913)	0.719	0.471	0.348	0.727	0.717
Integrated	0.828 (0.645–0.957)	0.906	0.727	0.727	0.727	0.943

AUC, area under the receiver operating characteristic curve; CI, confidence interval.

Pairwise comparisons among the clinical-imaging, radiomics, and integrated models are summarized in Table 4. In the external validation cohort, DeLong analysis indicated that the integrated model had a significantly higher AUC than the clinical-imaging model (*Z* = −2.495, *p* = 0.013) and the radiomics model (*Z* = −2.021, *p* = 0.043). Relative to the clinical-imaging model, the integrated model showed significant improvements in NRI (*p* = 0.020), IDI (*p* = 0.011), overall accuracy (*p* = 0.011), sensitivity (*p* = 0.031), and DCA (*p* = 0.014). Compared with the radiomics model, the integrated model also demonstrated significant improvements in NRI (*p* = 0.044), IDI (*p* = 0.026), accuracy (*p* = 0.034), sensitivity (*p* = 0.031), and DCA (*p* = 0.038).

**Table 4:** Comparative performance of radiomics, clinical-imaging, and integrated models for predicting any adverse progression in children with MPP.

Method	DeLong		NRI	IDI	McNemar				DCA
	*Z* value	*P* value	*P* value	*P* value	χ^2^	Acc *P*	Sens *P*	Spec *P*	*P* value
Training cohort									
Clinical-imaging vs. Radiomics	−4.706	< 0.001	< 0.001	< 0.001	19.360	< 0.001	< 0.001	1.000	< 0.001
Clinical-imaging vs. Integrated	−5.214	< 0.001	< 0.001	< 0.001	22.154	< 0.001	< 0.001	0.250	< 0.001
Radiomics vs. Integrated	−3.126	0.002	0.018	0.004	8.333	0.004	0.021	0.500	0.006
Testing cohort									
Clinical-imaging vs. Radiomics	−1.923	0.055	0.071	0.083	2.250	0.134	0.125	1.000	0.090
Clinical-imaging vs. Integrated	−2.734	0.006	0.012	0.009	7.143	0.008	0.021	0.500	0.011
Radiomics vs. Integrated	−2.118	0.034	0.029	0.018	5.333	0.021	0.031	1.000	0.024
Validation cohort									
Clinical-imaging vs. Radiomics	−1.691	0.091	0.108	0.074	2.667	0.102	0.125	1.000	0.080
Clinical-imaging vs. Integrated	−2.495	0.013	0.020	0.011	6.400	0.011	0.031	0.500	0.014
Radiomics vs. Integrated	−2.021	0.043	0.044	0.026	4.500	0.034	0.031	1.000	0.038

NRI, net reclassification improvement; IDI, integrated discrimination improvement; DCA, decision curve analysis; Acc, accuracy; Sens, sensitivity; Spec, specificity.

### Exploratory component-level performance according to adverse progression phenotype

Component-level performance results are summarized in Supplementary Table S4. In the training cohort, both the radiomics and integrated models correctly identified all cases across the three outcome phenotypes (sensitivity: 1.000 for each). In the testing cohort, the radiomics and integrated models each identified 0/3 RMPP-only cases, 0/1 PB-only case, and 2/6 concurrent RMPP/PB cases (sensitivities: 0.000, 0.000, and 0.333, respectively).

In the external validation cohort, the radiomics model identified 2/3 RMPP-only cases, 0/1 PB-only case, and 5/7 concurrent RMPP/PB cases, corresponding to sensitivities of 0.667, 0.000, and 0.714, respectively. The integrated model identified 3/3 RMPP-only cases, 0/1 PB-only case, and 5/7 concurrent RMPP/PB cases, corresponding to sensitivities of 1.000, 0.000, and 0.714, respectively.

### SHAP-based explanation of the integrated model

**Figure 4:**
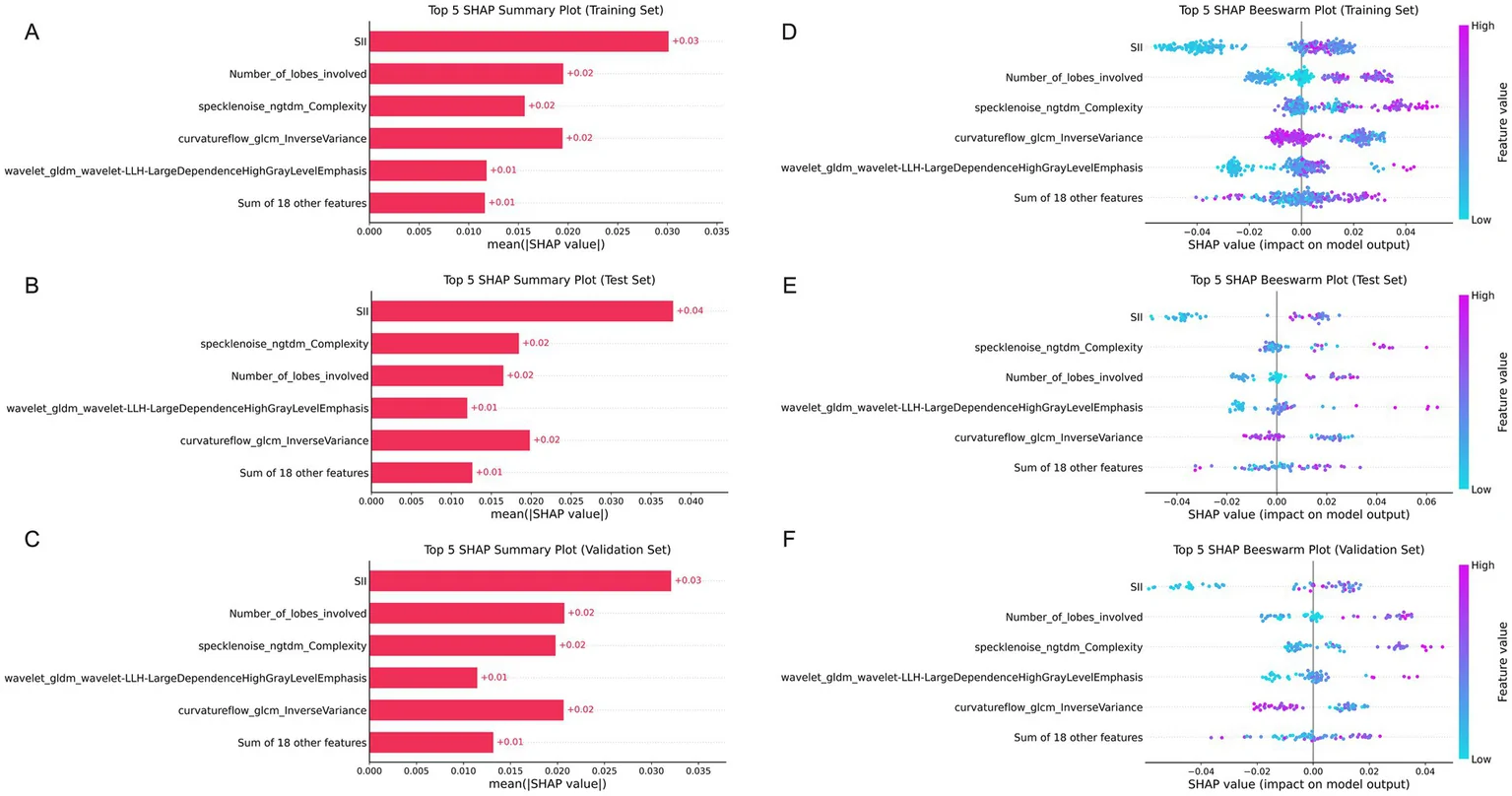
SHAP interpretation of the integrated model for predicting adverse progression in children with MPP. **(A–C)** Mean absolute SHAP values showing global feature importance in the training, test, and external validation cohorts. **(D–F)** SHAP beeswarm plots showing the direction and magnitude of feature effects on model output. SII was modeled as an untransformed continuous variable. SHAP, Shapley Additive Explanations; SII, systemic immune-inflammation index; NGTDM, neighboring gray-tone difference matrix; GLCM, gray-level co-occurrence matrix; GLDM, gray-level dependence matrix; LLH, low-low-high wavelet sub-band.

In the validation cohort, SII showed the highest mean absolute SHAP value, indicating the greatest contribution to the prediction of any adverse progression. The number of involved lung lobes ranked second, followed by the radiomics features specklenoise_ngtdm_Complexity, curvatureflow_glcm_InverseVariance, and wavelet-LLH_gldm_LargeDependenceHighGrayLevelEmphasis. (Figure 4C). The SHAP beeswarm plot further demonstrated the direction and magnitude of feature effects on model output (Figure 4F). Higher SII values were predominantly associated with positive SHAP values, corresponding to an increased model-predicted probability of any adverse progression, whereas lower SII values were mainly associated with negative SHAP values. Similarly, a greater number of involved lung lobes tended to contribute positively to the predicted risk. The radiomics features showed heterogeneous distributions of SHAP values, with both positive and negative contributions observed across different feature levels, reflecting their complementary roles in risk prediction.

## Discussion

In this multicenter retrospective study, we developed and externally validated an early risk stratification framework for adverse progression in children with MPP. The main finding is that a model integrating admission clinical-inflammatory indices, conventional CT features, and CT-derived radiomics provided robust and interpretable prediction of adverse progression, outperforming either clinical-imaging or radiomics information alone.

The dominant contribution of the SII suggests that host inflammatory dysregulation may be closely associated with the transition from uncomplicated MPP to progressive disease. SII integrates neutrophil expansion, platelet activation, and lymphocyte suppression, thereby capturing both innate immune activation and inflammation-associated thrombo-inflammatory status more comprehensively than isolated leukocyte indices. This interpretation is consistent with evidence that RMPP is not solely a consequence of pathogen persistence or macrolide resistance but is strongly shaped by excessive host immune responses, chemokine remodeling, and systemic inflammation (2, 4, 20). The associations of D-dimer and WBC count with adverse progression further support this biological axis. Elevated D-dimer reflects fibrin turnover and coagulation activation, which is particularly relevant in MPP complicated by airway casts, microvascular injury, and thromboembolic phenomena (7, 8). Thus, the laboratory predictors identified here should not be interpreted merely as nonspecific markers of infection, but rather as measurable surrogates of an inflammatory–coagulative milieu that may predispose to persistent fever, airway obstruction, and radiological deterioration.

Conventional CT findings provided complementary anatomical information. Lobar atelectasis and the number of involved lung lobes were consistently different between progression and non-progression groups across the training, testing, and external validation cohorts, suggesting that early structural disease burden is a stable marker of subsequent clinical deterioration. Lobar atelectasis in MPP can reflect mucus plugging, impaired mucociliary clearance, bronchial cast formation, and regional ventilation collapse, all of which are closely related to PB development and prolonged disease course. Similarly, multilobar involvement indicates a wider inflammatory field and greater bronchiolar or parenchymal injury, which may lower the threshold for refractory disease. These findings align with prior reports showing that consolidation/atelectasis phenotypes and CT-based severity scores are associated with more severe MPP, delayed recovery, and adverse pulmonary outcomes (11, 12). Nevertheless, the lower discriminative performance of the Clinical-Imaging model indicates that visually assessed CT features, although clinically intuitive, do not fully capture the spatial heterogeneity of pulmonary lesions.

Radiomics addressed this limitation by quantifying lesion heterogeneity beyond visual interpretation. The selected radiomics features, including NGTDM complexity, GLCM inverse variance, and GLDM high-gray-level dependence metrics, likely reflect variations in lesion texture, density distribution, and local structural organization caused by heterogeneous consolidation, inflammatory exudation, airway obstruction, and evolving parenchymal injury. These quantitative signatures may explain why the radiomics model achieved better external validation performance than the Clinical-Imaging model. The integrated model showed higher external-validation performance than the individual Clinical-Imaging and Radiomics models. This observation is consistent with the radiomics principle that image-derived phenotypes acquire maximal clinical value when embedded within a clinically meaningful prediction framework rather than used as isolated mathematical descriptors (13). However, these differences should be interpreted cautiously given the limited number of adverse-progression events.

Compared with previous studies, the present work extends the field in several aspects. Earlier prediction studies of RMPP or PB have mainly relied on clinical symptoms, routine laboratory indices, bronchoscopy findings, or conventional imaging variables (6, 21). More recent work has introduced CT scores, automated CT quantification, or radiomics for RMPP, delayed radiological recovery, and PB identification (12, 15–17). Our study differs from previous outcome-specific prediction studies by focusing on the composite endpoint of any adverse progression. This endpoint was selected as a pragmatic marker of clinically important deterioration rather than to imply that RMPP and PB are pathophysiologically, diagnostically, or therapeutically equivalent. Although RMPP primarily reflects persistent clinical or radiological nonresponse despite appropriate treatment and PB represents a bronchoscopically confirmed airway-obstructive complication, both outcomes may occur separately or concurrently and may prompt intensified surveillance, additional airway or imaging assessment, and escalation of management. The composite framework was therefore intended to address the shared early clinical decision of identifying children at risk of a subsequently complicated disease course. The use of an independent external validation cohort further strengthens the evidence for generalizability, while SHAP analysis improves model transparency by demonstrating that the integrated model was driven by clinically plausible variables rather than uninterpretable noise.

These findings suggest potential clinical utility for early risk stratification. Early identification of children at high risk for RMPP or PB may support intensified monitoring, earlier multidisciplinary assessment, timely consideration of bronchoscopy when airway obstruction is suspected, and individualized evaluation of anti-inflammatory or second-line antimicrobial strategies in accordance with guideline-based care (4, 20).

The relatively high specificity observed in the external validation cohort suggests that the model may help identify patients who warrant closer clinical assessment. However, because sensitivity was moderate, a low-risk prediction should not be used to exclude the possibility of subsequent progression. Alternative thresholds and resampling strategies were not evaluated, and although a lower threshold might improve sensitivity at the expense of specificity, the clinically appropriate trade-off requires prospective validation. Model estimates should therefore be interpreted together with the patient’s clinical course, laboratory findings, conventional imaging assessment, and established indications for bronchoscopy. Decisions regarding bronchoscopy, anti-inflammatory therapy, antimicrobial adjustment, or other treatment escalation should continue to follow clinical judgment and guideline-based care rather than being determined by the model output alone. The model is intended for children who have already undergone chest CT for established clinical indications and should not be used to justify routine CT screening in pediatric MPP. No additional CT examination should be performed solely to obtain a radiomics prediction, and CT use should remain governed by standard pediatric imaging protocols and individualized risk–benefit assessment.

This study has limitations. First, the retrospective design and requirement for clinically acquired CT may have introduced selection bias toward patients with greater clinical concern, limiting generalizability to unselected children with MPP, particularly those managed without CT; unmeasured confounding related to treatment timing, macrolide resistance, pathogen load, corticosteroid exposure, or bronchoscopy indication may also have influenced outcome classification. Second, although multicenter data and independent external validation were included, the external validation cohort contained only 11 adverse-progression events, resulting in wide confidence intervals and limited statistical power. DeLong, NRI, and IDI estimates may therefore be unstable and sensitive to a small number of observations, and the observed between-model differences should be interpreted as exploratory pending validation in larger cohorts. Third, manual lesion segmentation, despite good interobserver reproducibility, is time-consuming and may limit immediate clinical deployment. Future studies should evaluate automated or semi-automated segmentation pipelines and assess their stability across CT scanners and reconstruction protocols. Fourth, the composite endpoint included RMPP and PB, which overlap clinically but differ in pathophysiology, diagnosis, and management. The component groups were small and imbalanced (43 concurrent, 19 RMPP-only, 8 PB-only), with only one PB-only case in each held-out cohort. As a result, component-level estimates were imprecise and did not allow reliable comparisons or separate validation. The models should therefore be interpreted as predicting composite risk rather than RMPP- or PB-specific outcomes, and larger cohorts are needed for subtype-specific modeling. Finally, although the integrated model demonstrated superior discrimination, calibration, reclassification, and decision-curve performance compared with the radiomics and Clinical-Imaging models, this study did not test whether model-guided management improves patient outcomes. Prospective impact studies are therefore needed before routine implementation.

## Conclusion

In conclusion, the integrated model combining admission inflammatory indices, conventional CT findings, and CT-derived radiomics features showed potential for early risk stratification of adverse progression in pediatric MPP.

## Data Availability

The data analyzed in this study is subject to the following licenses/restrictions: the datasets generated during the current study are not publicly available due the hospital’s policies or confidentiality agreements but are available from the corresponding author on reasonable request. Requests to access these datasets should be directed to XY, jilly0075@163.com.
