# Supplementary material for: Early risk stratification of adverse progression in pediatric Mycoplasma pneumoniae pneumonia using a multicenter CT radiomics model
Source: Front Med (Lausanne). 2026 Aug 20;13:1905516. doi: 10.3389/fmed.2026.1905516 (PMC13538858; doi:10.3389/fmed.2026.1905516)
Supplement: Supplementary file 1 [file Table_1.DOCX]

**Supplementary information**

# 1. CT image acquisition parameters

Chest CT examinations were performed using three scanners with standardized lung-window reconstruction. For the United-Imaging UCT968 scanner, the parameters were: tube voltage, 120 kV; tube current, 20-50 mA; pitch, 0.984; matrix, 512 x 512; slice thickness, 5 mm; lung window, window width 1500 HU and window level -500 HU; mediastinal window, window width 350 HU and window level 40 HU; and axial lung-window reconstruction with a slice thickness of 0.625-1.250 mm. For the Philips Brilliance iCT scanner, the parameters were: tube voltage, 120 kV; tube current, 50-300 mA; pitch, 1; matrix, 512 x 512; slice thickness, 5 mm; lung window, window width 1500 HU and window level -500 HU; mediastinal window, window width 350 HU and window level 40 HU; and axial lung-window reconstruction with a slice thickness of 1.25 mm. For the Siemens SOMATOM Perspective scanner, the parameters were: detector collimation, 64 x 0.6 mm; tube voltage, 120 kV; adaptive tube-current modulation; matrix, 512 x 512; lung window, window width 1500 HU and window level -500 HU; mediastinal window, window width 350 HU and window level 40 HU; and axial lung-window reconstruction with a slice thickness of 1.25 mm. The CT acquisition parameters are summarized in Table S1.

**Table S1. CT image acquisition parameters**

| **CT scanner** | **320-detector CT scanner (UCT968)** | **256-detector CT scanner (SOMATOM Perspective)** | **64-detector CT scanner (Brilliance iCT)** |
| --- | --- | --- | --- |
| Center | Center A and B | Center A, B and C | Center C |
| Manufacturer | United-Imaging | SIEMENS | PHILIPS |
| Gantry rotation time (s) | 0.5 | 0.5 | 0.6 |
| Tube voltage (kV) | 120 | 120 | 120 |
| Tube current (mA) | 20-50 | 50-300 | Automatic |
| Matrix | 512 x 512 | 512 x 512 | 512 x 512 |
| Slice thickness for axial lung-window reconstruction (mm) | 0.625-1.250 | 1.250 | 1.250 |

*Abbreviations: s, second; kV, kilovolt; mA, milliampere; mm, millimetre; HU, Hounsfield unit.*

# 2. Radiomics filter implementation and reproducibility

Radiomics features were extracted from the manually segmented lesion ROIs after image preprocessing. Filtered image types were generated before feature extraction to capture intensity, edge, noise-perturbed, smoothing, and multiscale texture information. The same extraction configuration was applied to all training, testing, and validation images, whereas feature selection was performed only within the training cohort. The original software-manual descriptions of individual filters were replaced by the reproducibility checklist in Table S2. Unless otherwise specified in the extraction configuration, software-default parameter values were kept unchanged across all cohorts. All deterministic filters were applied once per image. For stochastic noise filters, the random seed was fixed before filtering to ensure repeatable feature values.

Feature classes and feature counts. From the original and transformed CT scans, a total of 1,904 radiomics features were generated by applying 15 filters. These features encompassed seven primary classes: first-order statistics (n=378), shape descriptors (n=14), gray-level co-occurrence matrix (GLCM; n=441), gray-level run-length matrix (GLRLM; n=336), gray-level size-zone matrix (GLSZM; n=336), gray-level dependence matrix (GLDM; n=294), and neighboring gray-tone difference matrix (NGTDM; n=105). Shape descriptors were calculated from the original ROI mask only, whereas first-order and texture features were extracted from the original and transformed image types. The same feature-extraction configuration was used for the training, testing, and validation cohorts. Feature selection was performed exclusively within the training cohort.

Table S2. CT image filters used for radiomics feature extraction and reproducibility reporting

| **Filter name** | **Implementation** | **Parameters to report or fixed in extraction configuration** | **Feature classes extracted** | **Feature number** | **Random seed / determinism** |
| --- | --- | --- | --- | --- | --- |
| Original image | Unfiltered CT image within the segmented ROI. | No image filter applied. | First-order, shape, GLCM, GLRLM, GLSZM, GLDM, NGTDM | Included in the total feature pool (n=1,904); shape descriptors (n=14) extracted from the original ROI mask only. | Deterministic |
| Additive Gaussian noise | Additive Gaussian white-noise perturbation of voxel intensities. | Noise mean; noise variance or standard deviation; intensity scaling; identical setting across cohorts. | First-order, GLCM, GLRLM, GLSZM, GLDM, NGTDM | Included in the total feature pool (n=1,904); class-wise counts are summarized in Table S3. | Stochastic; fixed random seed required |
| Bilateral filter | Edge-preserving smoothing based on spatial proximity and intensity similarity. | Spatial/domain sigma; intensity/range sigma; kernel or neighborhood setting. | First-order, GLCM, GLRLM, GLSZM, GLDM, NGTDM | Included in the total feature pool (n=1,904); class-wise counts are summarized in Table S3. | Deterministic |
| Binomial blur | Separable binomial smoothing applied along image dimensions. | Number of repetitions or kernel radius. | First-order, GLCM, GLRLM, GLSZM, GLDM, NGTDM | Included in the total feature pool (n=1,904); class-wise counts are summarized in Table S3. | Deterministic |
| Box mean filter | Fast rectangular moving-average filter. | Neighborhood radius or box/window size. | First-order, GLCM, GLRLM, GLSZM, GLDM, NGTDM | Included in the total feature pool (n=1,904); class-wise counts are summarized in Table S3. | Deterministic |
| Box sigma filter | Local sigma filter using a rectangular neighborhood. | Neighborhood radius or box/window size; sigma threshold if enabled. | First-order, GLCM, GLRLM, GLSZM, GLDM, NGTDM | Included in the total feature pool (n=1,904); class-wise counts are summarized in Table S3. | Deterministic |
| Curvature flow filter | Curvature-driven denoising that smooths homogeneous regions while preserving edges. | Number of iterations; time step. | First-order, GLCM, GLRLM, GLSZM, GLDM, NGTDM | Included in the total feature pool (n=1,904); class-wise counts are summarized in Table S3. | Deterministic |
| Discrete Gaussian filter | Gaussian smoothing by discrete separable convolution. | Gaussian variance or sigma; maximum kernel width; image-spacing option. | First-order, GLCM, GLRLM, GLSZM, GLDM, NGTDM | Included in the total feature pool (n=1,904); class-wise counts are summarized in Table S3. | Deterministic |
| Laplacian sharpening filter | Laplacian-based edge enhancement. | Kernel implementation; no stochastic parameter. | First-order, GLCM, GLRLM, GLSZM, GLDM, NGTDM | Included in the total feature pool (n=1,904); class-wise counts are summarized in Table S3. | Deterministic |
| Mean filter | Linear neighborhood averaging filter. | Neighborhood radius. | First-order, GLCM, GLRLM, GLSZM, GLDM, NGTDM | Included in the total feature pool (n=1,904); class-wise counts are summarized in Table S3. | Deterministic |
| Median filter | Nonlinear neighborhood median filter. | Neighborhood radius. | First-order, GLCM, GLRLM, GLSZM, GLDM, NGTDM | Included in the total feature pool (n=1,904); class-wise counts are summarized in Table S3. | Deterministic |
| Normalize filter | Intensity standardization to zero mean and unit variance. | Mean and standard deviation computed from the image; no external tunable parameter unless scaling is specified. | First-order, GLCM, GLRLM, GLSZM, GLDM, NGTDM | Included in the total feature pool (n=1,904); class-wise counts are summarized in Table S3. | Deterministic |
| Shot-noise filter | Poisson-distributed intensity-dependent noise perturbation. | Scale or gain factor; Poisson-to-Gaussian approximation threshold if modified. | First-order, GLCM, GLRLM, GLSZM, GLDM, NGTDM | Included in the total feature pool (n=1,904); class-wise counts are summarized in Table S3. | Stochastic; fixed random seed required |
| Smoothing recursive Gaussian filter | Recursive Gaussian smoothing applied to the image. | Gaussian sigma; normalization-across-scale setting if enabled. | First-order, GLCM, GLRLM, GLSZM, GLDM, NGTDM | Included in the total feature pool (n=1,904); class-wise counts are summarized in Table S3. | Deterministic |
| Speckle-noise filter | Multiplicative Gamma-distributed noise perturbation proportional to voxel intensity. | Noise standard deviation or noise level; intensity scaling if applied. | First-order, GLCM, GLRLM, GLSZM, GLDM, NGTDM | Included in the total feature pool (n=1,904); class-wise counts are summarized in Table S3. | Stochastic; fixed random seed required |
| Wavelet filter | First-level 3D wavelet decomposition into eight sub-band images. | Wavelet basis; decomposition level = 1; sub-bands LLL, LLH, LHL, LHH, HLL, HLH, HHL, and HHH. | First-order, GLCM, GLRLM, GLSZM, GLDM, NGTDM | Included in the total feature pool (n=1,904); class-wise counts are summarized in Table S3. | Deterministic |

Abbreviations: ROI, region of interest; GLCM, gray-level co-occurrence matrix; GLRLM, gray-level run-length matrix; GLSZM, gray-level size-zone matrix; GLDM, gray-level dependence matrix; NGTDM, neighboring gray-tone difference matrix. Exact numeric values of tunable parameters should match the executed extraction configuration file and should not be changed between cohorts.

Table S3. Hyperparameter search space for random forest models.

| Hyperparameter | Candidate values | Clinical-Imaging model | Radiomics model | Integrated model |
| --- | --- | --- | --- | --- |
| n_estimators | 200, 500, 1000 | 500 | 1000 | 1000 |
| max_depth | None, 3, 5, 10, 20 | 5 | 5 | 5 |
| min_samples_split | 2, 5, 10 | 10 | 10 | 10 |
| min_samples_leaf | 1, 2, 4, 8 | 4 | 8 | 8 |
| max_features | "sqrt", "log2", 0.3, 0.5 | "sqrt" | 0.3 | 0.3 |
| bootstrap | True, False | True | True | True |
| class_weight | None, "balanced", "balanced_subsample" | "balanced" | "balanced_subsample" | "balanced_subsample" |
| Optimization metric | Mean cross-validated AUC | Mean AUC | Mean AUC | Mean AUC |
| Cross-validation strategy | Stratified five-fold cross-validation | Five-fold | Five-fold | Five-fold |
| Classification threshold | Prespecified fixed threshold | 0.50 | 0.50 | 0.50 |
| Random seed | Fixed integer | 42 | 42 | 42 |

AUC, area under the receiver operating characteristic curve; CT, computed tomography.

Supplementary Table S4. Exploratory component-level performance of the Clinical-Imaging, Radiomics, and Integrated models

| Cohort | Model | Outcome component | n | Median predicted probability (IQR) | Sensitivity (95% exact CI) |
| --- | --- | --- | --- | --- | --- |
| Training | Clinical-Imaging | RMPP only | 13 | 0.930 (0.857–0.970) | 1.000 (0.753–1.000) |
| Training | Clinical-Imaging | PB only | 6 | 0.780 (0.704–0.922) | 0.833 (0.359–0.996) |
| Training | Clinical-Imaging | Concurrent RMPP/PB | 30 | 0.848 (0.701–0.949) | 0.900 (0.735–0.979) |
| Training | Radiomics | RMPP only | 13 | 0.853 (0.750–0.903) | 1.000 (0.753–1.000) |
| Training | Radiomics | PB only | 6 | 0.713 (0.701–0.734) | 1.000 (0.541–1.000) |
| Training | Radiomics | Concurrent RMPP/PB | 30 | 0.754 (0.670–0.804) | 1.000 (0.884–1.000) |
| Training | Integrated | RMPP only | 13 | 0.802 (0.758–0.871) | 1.000 (0.753–1.000) |
| Training | Integrated | PB only | 6 | 0.680 (0.671–0.702) | 1.000 (0.541–1.000) |
| Training | Integrated | Concurrent RMPP/PB | 30 | 0.732 (0.635–0.778) | 1.000 (0.884–1.000) |
| Testing | Clinical-Imaging | RMPP only | 3 | 0.714 (0.431–0.809) | 0.667 (0.094–0.992) |
| Testing | Clinical-Imaging | PB only | 1 | 0.018 (0.018–0.018) | 0.000 (0.000–0.975) |
| Testing | Clinical-Imaging | Concurrent RMPP/PB | 6 | 0.547 (0.323–0.687) | 0.500 (0.118–0.882) |
| Testing | Radiomics | RMPP only | 3 | 0.208 (0.155–0.341) | 0.000 (0.000–0.708) |
| Testing | Radiomics | PB only | 1 | 0.146 (0.146–0.146) | 0.000 (0.000–0.975) |
| Testing | Radiomics | Concurrent RMPP/PB | 6 | 0.369 (0.318–0.546) | 0.333 (0.043–0.777) |
| Testing | Integrated | RMPP only | 3 | 0.232 (0.216–0.331) | 0.000 (0.000–0.708) |
| Testing | Integrated | PB only | 1 | 0.159 (0.159–0.159) | 0.000 (0.000–0.975) |
| Testing | Integrated | Concurrent RMPP/PB | 6 | 0.410 (0.297–0.540) | 0.333 (0.043–0.777) |
| External validation | Clinical-Imaging | RMPP only | 3 | 0.923 (0.726–0.955) | 1.000 (0.292–1.000) |
| External validation | Clinical-Imaging | PB only | 1 | 0.786 (0.786–0.786) | 1.000 (0.025–1.000) |
| External validation | Clinical-Imaging | Concurrent RMPP/PB | 7 | 0.562 (0.257–0.970) | 0.571 (0.184–0.901) |
| External validation | Radiomics | RMPP only | 3 | 0.743 (0.509–0.756) | 0.667 (0.094–0.992) |
| External validation | Radiomics | PB only | 1 | 0.180 (0.180–0.180) | 0.000 (0.000–0.975) |
| External validation | Radiomics | Concurrent RMPP/PB | 7 | 0.544 (0.459–0.589) | 0.714 (0.290–0.963) |
| External validation | Integrated | RMPP only | 3 | 0.635 (0.516–0.711) | 0.667 (0.094–0.992) |
| External validation | Integrated | PB only | 0 | 0.218 (0.218–0.218) | 0.000 (0.000–0.975) |
| External validation | Integrated | Concurrent RMPP/PB | 5 | 0.585 (0.479–0.595) | 1.000 (0.292–1.000) |

Data are presented for refractory Mycoplasma pneumoniae pneumonia only, plastic bronchitis only, and concurrent cases. Predicted probabilities are shown as medians (interquartile ranges), with ≥0.50 defined as a positive prediction. Correctly identified cases are expressed as true positives over total cases, and sensitivity is reported with exact 95% confidence intervals (Clopper–Pearson method). Analyses were exploratory without model retraining or threshold adjustment. Due to small and imbalanced sample sizes, especially for plastic bronchitis-only cases, results are imprecise and no formal comparisons were performed.

CI, confidence interval; IQR, interquartile range; MPP, Mycoplasma pneumoniae pneumonia; PB, plastic bronchitis; RMPP, refractory Mycoplasma pneumoniae pneumonia.
